# Prognostic impact of tumor spread through air spaces for T2aN0 stage IB non‐small cell lung cancer

**DOI:** 10.1002/cam4.6211

**Published:** 2023-06-06

**Authors:** Zixuan Chen, Xianqiao Wu, Tianzheng Fang, Zhen Ge, Jiayuan Liu, Qinglong Wu, Lin Zhou, Jianfei Shen, Chengwei Zhou

**Affiliations:** ^1^ Thoracic Surgery Department The First Affiliated Hospital of Ningbo University Ningbo China; ^2^ Cardiothoracic Surgery Department Taizhou Hospital of Zhejiang Province, Wenzhou Medical University Linhai China

**Keywords:** IB stage non‐small cell lung cancer, overall survival, recurrence‐free survival, spread through air spaces

## Abstract

**Background:**

Spread through air spaces (STAS) is a pattern of invasion recently identified in non‐small cell lung cancer (NSCLC), with a poor prognosis. However, the predictive impact of STAS in stage IB NSCLC is not well understood. This investigation aims to assess the prognostic influence of STAS in stage IB NSCLC.

**Methods:**

We reviewed 130 resected stage IB NSCLC between 2010 and 2015. Beyond the central tumor edge, lung parenchymal air gaps containing cancer cells were identified as STAS. In order to estimate recurrence‐free survival (RFS) and overall survival (OS), Cox models and Kaplan–Meier techniques were utilized. Logistic regression analysis was employed to define the factors influencing STAS.

**Results:**

Of 130 patients, 72 (55.4%) had STAS. STAS was a significant prognosticator. Kaplan–Meier method showed that STAS‐positive patients had a significantly lower OS and RFS than STAS‐negative patients (5‐year OS, 66.5% vs. 90.4%, *p* = 0.02; 5‐year RFS, 59.5% vs. 89.7%, *p* = 0.004) In a semiquantitative assessment, the RFS and OS were shorter in survival analysis when STAS increased (5‐year RFS, 89.7%, no STAS, 61.8%, low STAS, 57.2%, high STAS, *p* = 0.013; 5‐year OS, 90.4%, no STAS, 78.3%, low STAS, 57.2%, high STAS, *p* = 0.002). The association between STAS and poor differentiation, adenocarcinoma, and vascular invasion (*p* value was <0.001, 0.047, and 0.041, respectively) was statistically significant.

**Conclusions:**

The STAS is an aggressive pathological feature. RFS and OS could be significantly reduced by STAS, while it also serves as an independent predictor.

## 1 INTRODUCTION

A novel tumor invasion method called spread through air spaces (STAS) has been discovered.^1^ There are three morphological features for STAS, which refers to tumor cells that are located inside the air gaps of the pulmonary parenchyma beyond the border of the primary cancer: single cells, solid nests, and micropapillary clusters.^2^ Based on previous studies, STAS worsens survival and increases recurrence risks in individuals with postoperative non‐small cell lung cancer (NSCLC). Additionally, there is evidence that STAS is an independent predictive indicator.^2^, ^3^, ^4^, ^5^

By analyzing sequences of 411 stage IA pulmonary adenocarcinomas (ADC), Kadota et al. discovered that STAS can reduce overall survival (OS) by 5 years and increase cumulative incidence of recurrence (CIR) by 5 years as well, in postoperative patients. Furthermore, STAS was discovered to be a substantial risk factor for recurrence with limited resection.^2^ Similarly, Warth et al. observed that STAS can reduce disease‐free survival (DFS) as well as OS. According to a multivariate study, STAS is a substantial independent risk factor for prognosis.^6^ In addition to lung adenocarcinoma, the STAS has also been identified in pulmonary squamous cell carcinoma (SCC) and small cell lung cancer.^7^, ^8^ STAS was demonstrated to be a major predictive factor in SCC in multiple studies conducted in 2017.^8^, ^9^ STAS was semiquantitatively examined by Uruga et al. in 208 stages IA ADC. STAS was classified arbitrarily in the present investigation as low or high, with the high STAS being related to poorer recurrence‐free survival (RFS).^10^ Several studies have focused on the negative effect of stage IA NSCLC on survival and recurrence,^5^, ^10^, ^11^ but there are few reports on stage IB NSCLC. To test STAS influence on prognosis, retrospective analyses were conducted on clinical data from patients experiencing surgical stage IB lung cancer resection in this study.

## 2 MATERIALS AND METHODS

### 2.1 Patients

Institutional ethic review boards of The First Affiliated Hospital of Ningbo University and Taizhou Hospital of Zhejiang Province have approved this retrospective study (Project batch number: KS20232003; Project acceptance number: 2023KS0009), which also included the clinical databases that we reviewed from 2010 to 2015 for lung cancer patients with surgical resection. American Joint Committee on Cancer (AJCC) staging 8th version guidelines was used to identify the pathologic phase.^12^ The following were the inclusion criteria: (1) age from 18 to 80; (2) The postoperative pathological diagnosis of NSCLC with stage T2aN0M0; (3) radical surgical resection (R0 resection); (4) no preoperative systemic chemoradiotherapy. Conversely, the following were the exclusion criteria: (1) incomplete patient clinical data; (2) incomplete multiple lung nodules resection; (3) positive surgical margins; (4) no serious diseases on vital organs such as liver, kidney, and heart. We identified a total of 130 cases using these criteria.

### 2.2 Histologic evaluation

Typically, 130 Eosin‐stained tumor and hematoxylin slides were examined by two pathologists blinded to the participant's medical outcome using a Leica DM2000 microscope (Leica Microsystems Inc. Vizsla, Germany) and a typical 22‐mm diameter eyepiece.

Pathological evaluations were depending on the 2015 World Health Organization (WHO) categorization of pulmonary cancers and the AJCC TNM Classification, 8th edition. The STAS was identified as per the procedure by Kadota et al.^2^ There are three morphological patterns for STAS, which refers to cancerous cells that are located inside the air gaps of the pulmonary parenchyma beyond the border of the primary tumor: (1) solid nests or tumor islands made up of solid cancer cells collections filling air gaps; (2) single cells made up of dispersed discohesive single cells; and (3) micro papillary constructions, which are papillary constructions without central fibrovascular centers that occasionally establish ring‐like structures within air spaces.^1^, ^2^ In the most prominent region, STAS was assessed semiquantitatively, according to Uruga et al.^10^ The STAS was classified into three categories: high STAS (≥5 clusters of STAS), low STAS (1–4 clusters of the micropapillary or solid nest–predominant STAS), and no STAS.^10^

### 2.3 Statistical analyses

The primary observation indicators of this study were OS and RFS.

The statistical software SPSS 21.0 was utilized for analysis. Quantitative data were evaluated using a *t*‐test, while qualitative data were examined using chi‐square and Fisher's exact tests. Log‐rank testing was performed to assess statistical variations, and Kaplan–Meier analysis was utilized to determine OS and RFS rates. Employing multivariate and univariate Cox proportional hazards regression models, the predictive factors were examined. The risk factors associated with STAS were predicted using the binary logistic regression model. Statistical significance is demonstrated by a *p* value less than 0.05.

## 3 RESULTS

### 3.1 Patient characteristics

**FIGURE 1:**
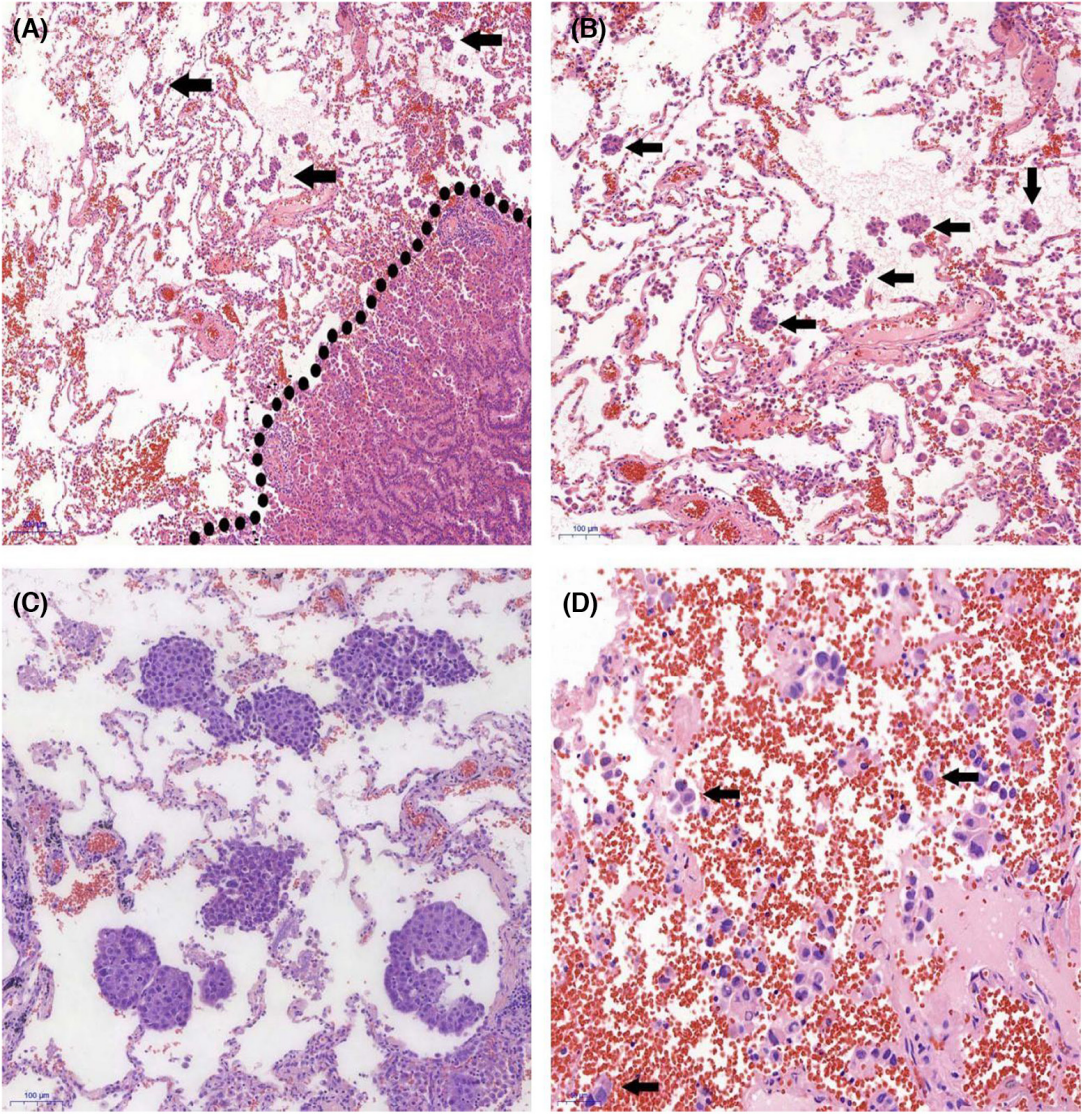
The histopathological feature of STAS (original magnification: ×5 in A; ×10 in B and C;×20 in D). (A) STAS was described as tumor cells within air spaces in the lung parenchyma beyond the edge of the main tumor； (B) Micropapillary pattern STA; (C) Soild pattern STAS; (D) Single cell pattern STAS.

From 2010 to 2015, 1149 NSCLC patients received surgical treatment at The First Affiliated Hospital of Ningbo University and Taizhou Hospital of Zhejiang Province. There were 142 patients with stage IB based on postoperative pathological staging. Finally, 130 patients with stage IB lung cancer (88 ADC and 42 SCC) met the criteria. All patients underwent VATS including uniport (55 cases) and multiport (75 cases) thoracoscopic surgery. One hundred and twenty patients underwent radical lobectomy, while 10 patients received limited resection. There were 30 patients received postoperative adjuvant chemotherapy, and 100 patients did not received postoperative adjuvant chemotherapy. Typically, 86 males and 44 females were included, with a mean age of 64.12 years, ranging from 31 to 80 years. Sixty‐three patients had never smoked while 67 patients were past or current smokers. There were 72 STAS‐positive cases (micropapillary clusters in 22 cases, solid nests in 46 cases, and single cells in 4 cases), and 58 negative cases. STAS was found to be low in 33 cases and high in 39 cases. After surgery, the enrolled lung cancer patients received regular outpatient or telephone follow‐ups. The last follow‐up was on August 31, 2018, whereas the average follow‐up period was 46.89 (0.5–98) months. Twenty‐four patients died due to various reasons. In addition, 26 patients had tumor recurrence. Table 1 shows the demographic information of the enrolled patients. Figure 1 shows morphological features of STAS.

**TABLE 1:** Patient characteristics.

Variables	*N* = 130 (%)	Variables	*N* = 130 (%)
Gender		Histologic subtype	
Male	86 (66.2)	Basaloid	3 (2.3)
Female	44 (33.8)	Keratinizing	5 (3.8)
Age, years (mean, range)	64.12 (31–80)	Nonkeratinizing	34 (26.2)
≤64	63 (48.4)	Acinar	45 (34.6)
>64	67 (51.6)	Papillary	10 (7.7)
Smoking		Micropapillary	10 (7.7)
Former/current	67 (51.6)	Soild	15 (11.5)
Never	63 (48.4)	Lepidic	8 (6.2)
Surgery		Visceral pleural invasion	
Lobectomy	120 (92.3)	Yes	61 (46.9)
Limited resection	10 (7.7)	NO	69 (53.1)
Histologic type		Vascular invasion	
ADC	88 (67.7)	Yes	15 (11.5)
SCC	42 (32.3)	No	115 (88.5)
STAS		Histologic grade	
Positive	72 (55.4)	Well	4 (3.1)
Negative	58 (44.6)	Moderately	76 (58.5)
STAS quantity		Poorly	50 (38.4)
No	58 (44.6)	VATS port	
Low	33 (25.4)	Uniport	55 (42.3)
High	39 (30)	Multiport	75 (57.7)
Chemotherapy		Death	24 (18.5)
Yes	30 (23.1)	Tumor recurrence	26 (20)
No	100 (76.9)	Follow‐up time (month)	46.89

### 3.2 Association between clinical factors and STAS

A statistically significant relation was detected between STAS and vascular invasion, poor differentiation, and adenocarcinoma (*p* value was 0.041, <0.001, 0.047, respectively) (Table 2). Univariate regression analysis exhibited that STAS was strongly related to worse differentiation and vascular invasion (*p* value was <0.001 and 0.05, respectively). (Table 3) Besides, multivariate regression analysis showed that poor differentiation was an independent predictor of STAS (*p* = 0.002) (Table 3).

**TABLE 2:** The correlation between STAS and patient characteristics.

Variables	Total *N* = 130	STAS positive (*N* = 72)	STAS negative (*N* = 58)	*p*
Gender *n* (%)				
Male	86 (66.2)	47 (54.7)	39 (45.3)	0.814
Female	44 (33.8)	25 (56.8)	19 (43.2)	
Age, years *n* (%)				
≤64	63 (48.4)	37 (58.7)	26 (41.3)	0.457
>64	67 (51.6)	35 (52.2)	32 (47.8)	
Smoking *n* (%)				
Former/current	67 (51.6)	35 (52.2)	32 (47.8)	0.457
Never	63 (48.4)	37 (58.7)	26 (41.3)	
Histologic grade *n* (%)				
Well, moderately	79 (61.6)	32 (40.5)	47 (59.5)	<0.001^a^
Poorly	51 (38.4)	40 (78.4)	11 (21.6)	
Vascular invasion *n* (%)				
Yes	15 (11.5)	12 (80)	3 (20)	0.041^a^
No	115 (88.5)	60 (52.2)	55 (47.8)	
Visceral pleural invasion *n* (%)				
Yes	61 (46.9)	34 (55.7)	27 (44.3)	0.939
No	69 (53.1)	38 (55.1)	31 (44.9)	
Histologic type *n* (%)				
ADC	88 (67.7)	54 (61.4)	34 (38.6)	0.047^a^
SCC	42 (32.3)	18 (42.9)	24 (57.1)	

^a^ Significant *p* values *p* < 0.05.

**TABLE 3:** Univariate and multivariate analyses of factors associated with STAS.

Factors	Univariate analyses		Multivariate analyses	
	OR (95% CI)	*p*	OR (95% CI)	*p*
ADC	2.84 (0.96–8.35)	0.058	–	–
Visceral pleural invasion	0.57 (0.14–2.42)	0.449	–	–
Vascular invasion	4.49 (1.00–20.71)	0.050	3.88 (0.97–15.53)	0.055
Male	1.85 (0.55–6.19)	0.319	–	–
Smoking	0.55 (0.18–1.65)	0.284	–	–
Histologic grade poorly	5.77 (2.40–13.90)	<0.001^a^	3.97 (1.69–9.31)	0.002^a^
Age > 64 years	1.07 (0.48–2.40)	0.866	–	–

^a^ Significant *p* values *p* < 0.05.

### 3.3 Relationship between OS and STAS

**FIGURE 2:**
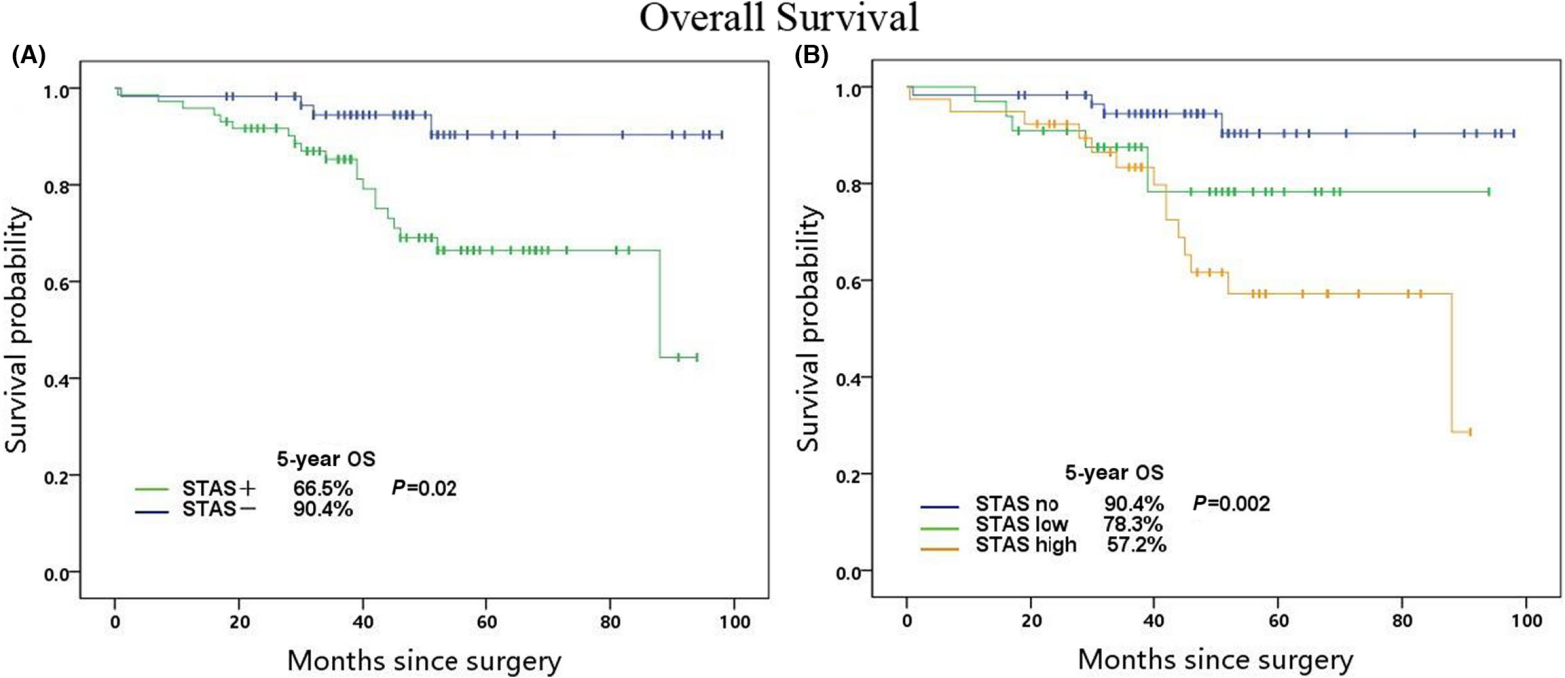
Kaplan‐Meier plot of 5‐yr Overall Survival stratified by STAS; (A) 5‐yr Overall Survival according to the presence of STAS; (B) 5‐yr Overall Survival according to the quantitativeness of STAS.

The 130 patients were monitored for an average of 46.89 months (ranging from 0.5 to 98 months), and 24 patients were died. Survival analysis showed that STAS‐positive patients had a significantly lower overall survival than STAS‐negative patients (66.5% vs. 90.4%, *p* = 0.02) (Figure 2A). Univariate survival analysis revealed that STAS, visceral pleural invasion, localized resection, vascular invasion, and poor differentiation were identified as risk factors impacting the patients' prognosis. Furthermore, multivariate survival analysis demonstrated that STAS was an independent risk factor for the prognosis of stage IB NSCLC (*p* = 0.027), and could lower the postoperative survival time (Table 4).

**TABLE 4:** Univariate and multivariate analyses of OS.

Variables	Univariate		Multivariate	
	HR (95% CI)	*p*	HR (95% CI)	*p*
STAS:positive vs. negative	4.59 (1.56–13.48)	0.006^a^	3.56 (1.16–10.97)	0.027^a^
Age: >64 vs. ≤64	1.46 (0.65–3.28)	0.364	–	–
Gender: male vs. female	0.70 (0.31–1.59)	0.397	–	–
Smoking: former/current vs. Never	1.32 (0.59–2.97)	0.501	–	–
ADC vs. SCC	0.94 (0.40–2.22)	0.894	–	–
Lobectomy vs. limited resection	0.30 (0.11–0.81)	0.017^a^	0.37 (0.13–1.10)	0.074
Visceral pleural invasion: yes vs. no	2.98 (1.17–7.54)	0.022^a^	2.01 (0.42–9.57)	0.380
Vascular invasion: yes vs. no	3.40 (1.34–8.66)	0.01^a^	1.67 (0.59–4.76)	0.335
Histologic grade: poorly vs. well, moderately	2.31 (1.03–5.17)	0.042^a^	1.78 (0.74–4.28)	0.200
Chemotherapy: yes vs. no	0.58 (0.23–1.47)	0.252	–	–

Abbreviations: CI, confidence interval; HR, hazard ratio.

^a^ Significant *p* values *p* < 0.05.

Participants in the high STAS group had a significantly poorer prognosis than those in the low STAS and STAS‐negative groups, according to the survival analysis of the STAS quantitative stratified group. There was a significant variation in the prognosis between the groups (5‐year OS rate of high STAS vs. low STAS vs. STAS negative: 57.2%, 78.3%, and 90.4%, *p* = 0.002, see Figure 2B).

### 3.4 Relationship between RFS and STAS

**FIGURE 3:**
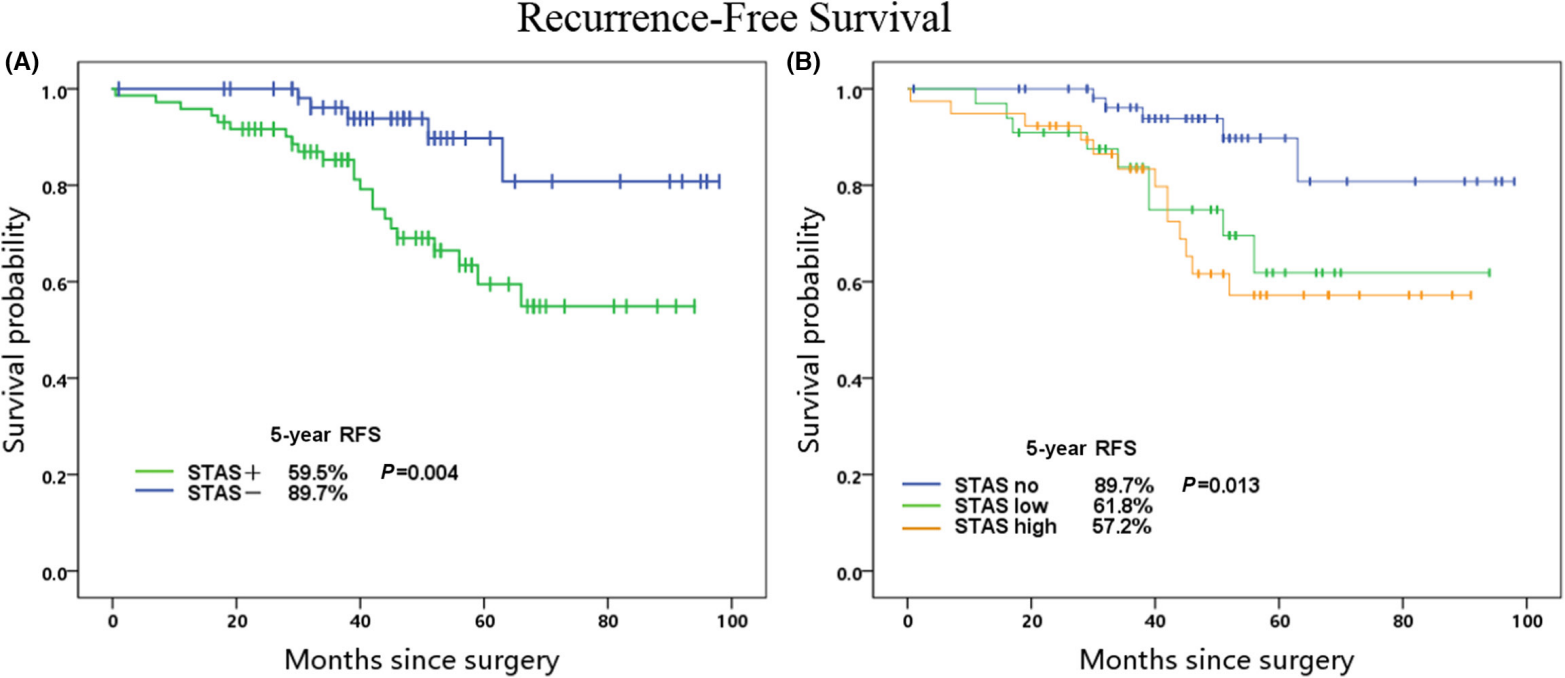
Kaplan‐Meier plot of 5‐yr Recurrence‐Free Survival stratified by STAS; (A) 5‐yr Recurrence‐Free Survival according to the presence of STAS; (B) 5‐yr Recurrence‐Free Survival according to the quantitativeness of STAS.

Twenty‐six (20%) of the 130 patients had tumor recurrences after surgery, twenty‐one of whom were STAS positive and five of whom were STAS negative. The STAS‐positive group had a recurrence rate of 29.2% (21/72) while the negative group had 8.6% (5/58). The STAS‐positive patients had a significantly lower 5‐year RFS than STAS‐negative patients (59.5% vs. 89.7%, *p* = 0.004, Figure 3A). In univariate survival analysis, vascular invasion (*p* = 0.022), poor differentiation (*p* = 0.021), and STAS (*p* = 0.007) were important factors for RFS; while multivariate survival analysis, STAS (*p* = 0.041) and vascular invasion (*p* = 0.041) were significant risk factors for RFS (Table 5).

**TABLE 5:** Univariate and multivariate analyses of RFS.

Variables	Univariate		Multivariate	
	HR (95% CI)	*p*	HR (95% CI)	*p*
STAS: positive vs. negative	3.82 (1.44–10.07)	0.007^a^	2.88 (1.04–7.96)	0.041^a^
Age:>64 vs. ≤64	1.53 (0.71–3.31)	0.275	–	–
Gender: male vs. female	0.71 (0.33–1.54)	0.391	–	–
Smoking: former/current vs. Never	1.37 (0.64–2.94)	0.413	–	–
ADC vs. SCC	0.92 (0.41–2.04)	0.829	–	–
Lobectomy vs. Limited resection	0.33 (0.12–0.88)	0.028^a^	0.45 (0.16‐1.28)	0.136
Visceral pleural invasion: yes vs. no	0.67 (0.31–1.46)	0.316	–	–
Vascular invasion: yes vs. no	2.90 (1.16–7.21)	0.022^a^	2.65 (1.04‐6.71)	0.041^a^
Histologic grade: poorly vs. well, moderately	2.44 (1.14–5.23)	0.021^a^	1.89 (0.85‐4.20)	0.117
Chemotherapy: yes vs. no	0.82 (0.36–1.82)	0.627	–	–

Abbreviations: CI, confidence interval; HR, hazard ratio.

^a^ Significant *p* values *p* < 0.05.

The recurrence‐free survival analysis in the STAS quantitative stratification group revealed that the 5‐year RFS of patients in the high STAS group was significantly lesser than that of the low STAS group and the STAS‐negative group. There were significant alterations in the prognosis between the groups (5‐year RFS high STAS vs. low STAS vs. low STAS vs. STAS negative: 57.2%, 61.8%, and 89.7%, *p* = 0.013) (Figure 3B).

## 4 DISCUSSION

Surgical resection is the recommended form of treatment for lung cancer, which is the primary reason for cancer‐related deaths globally.^13^ However, approximately 20%–40% of early‐stage NSCLC patients relapsed after surgery.^14^ Multiple pathological factors influence prognosis, such as histological subtype, lymphatic invasion, vascular invasion, and pleural invasion. Recently, researchers have discovered that STAS affects the prognosis of lung cancer.

In 2015, Kadota et al. defined STAS.^2^ In the same year, WHO added STAS as a new pattern of tumor invasiveness of ADC to the lung cancer guidelines.^1^ Since then, many studies have demonstrated that STAS can reduce the postoperative survival rate and increase the risk of postoperative recurrence, which is an important risk factor for NSCLC.^2^, ^4^, ^5^, ^8^, ^10^ However, in contemporary STAS‐related studies, early‐stage NSCLC (stage I) is the primary research object, with stage IA being the common one.^5^, ^10^, ^15^, ^16^ In our study, stage IB NSCLC was used as the research object, with the analysis results being consistent with the previous studies. Survival analysis showed that the OS rate and RFS rate of STAS‐positive patients were significantly lesser than those of STAS‐negative patients. Additionally, the semi‐quantitative analysis showed that the more prevalent the STAS is, the lower the OS rate and RFS rate of NSCLC.

The STAS is a newly discovered pathomorphological feature in NSCLC with a positivity rate ranging from 14.8% to 55.4%.^8^, ^10^, ^15^, ^17^ The positivity rate of STAS in small cell lung cancer is as high as 83%.^7^ We found the STAS positivity rate to be 55.4% in our study. According to Warth et al, the positive rate of STAS increased with advanced pathological stage.^6^ The previous research objects were mainly staged T1N0M0 NSCLC; however, in our study, stage T2aN0M0 NSCLC was the research object. This could explain the higher positive rate of STAS in this study. Additionally, STAS was significantly related to vascular invasion, pleural invasion, pathological differentiation, and gene mutations.^2^, ^6^, ^10^ Kadota et al. observed that in a series of 411 ADC, STAS was substantially correlated with visceral pleural invasion, vascular invasion, micropapillary pattern, and solid pattern.^2^ The STAS is linked with pathological stage, lymph node metastasis, and vascular invasion in SCC.^9^ Some researchers found that STAS was related to a reduced EGFR mutation rate and a high BRAF mutation rate, but not with KRAS mutation.^6^ Poor differentiation, vascular invasion, and lung adenocarcinoma were significant associated factors for STAS in our study, and poor differentiation was an independent predictor of STAS. in the multivariate binary regression analysis.

In their 2015 research report, Kadota et al. pointed out that in early‐stage ADC, STAS was a substantial risk factor for subjects with limited resection prognosis, but had no significant effect on patients who received lobectomy.^2^ Similarly, Eguchi et al. found that for individuals with STAS in stage T1N0M0 ADC, lobectomy was better than sublobar resection.^16^ Conversely, several studies have found that STAS adversely affects the prognosis of patients after lobectomy.^8^, ^9^, ^17^, ^18^ Eguchi et al. reported that 5 year‐CIR and 5 year‐CID of STAS‐positive were significantly higher than STAS‐negative (CIR: 15.2% vs. 7.8%; CID: 7.2% vs. 2.9%) in early‐stage ADC patients after lobectomy.^18^ After excluding limited resection, Shiono et al. discovered that STAS was an independent risk factor for lobectomy.^17^ According to Lu et al., STAS is an independent risk factor for lobectomy, and while it can reduce OS in limited resection, it is not an independent risk factor.^8^ Although 92.3% (120/130) of patients in our study underwent lobectomy, STAS had a significant effect on RFS and OS. This could imply that STAS is an independent predictive risk factor of the surgical approach; however, this hypothesis needs further research to verify.

In 2015, the adoption of STAS for ADC in the WHO categorization for lung cancer after verification in two large independent cohorts from Germany and the United States.^1^, ^2^, ^6^ Subsequent studies have confirmed and extended STAS.^19^ Numerous independent studies have confirmed that STAS is a novel morphological feature with important predictive value. Therefore, STAS should be involved in pathology reports to assist surgeons in evaluating prognosis and guiding postoperative treatment. It is still unclear, nevertheless, whether STAS represents an in vivo influence or a potential artifact.^20^ On the one hand, Blaauwgeers et al. conducted a multicenter study on lung cancer specimens that were not treated with formalin and discovered that as the number of incisions increased, so did the quantity of free cancer cell clusters in the alveolar space. As a result, they proposed that the occurrence of STAS could be linked to the processing of nonfixed lesions by pathologists.^21^, ^22^ Conversely, Lu et al. discovered STAS in the uncut tissue of fixed lesions.^23^ Several studies have confirmed the existence of tumor cell islands (STAS) in the alveolar space by 3D reconstruction of formalin‐fixed pathological sections.^24^ Further investigation is therefore required to understand the etiology of STAS. In addition to the pathology, other possible causes of STAS should be fully investigated. The mainstream surgical method for lung cancer is video‐assisted thoracic surgery (VATS) and radical resection.^25^, ^26^ After the tumor tissue has been excised, it is removed through a narrow hole in the chest wall. The compression of the diseased tissue during this process may result in the spread of tumor cells. Small tumor lesions are difficult to detect with the naked eye. Surgeons usually use their hands to touch and squeeze the lung lobes to locate lesions, which can potentially cause STAS.^19^ The majority of STAS‐related studies have confirmed that STAS is an important prognostic feature, and has a greater impact on limited resection than lobectomy.^2^, ^16^ Therefore, if intraoperative frozen sections could detect the presence or absence of STAS, it would help surgeons in choosing a surgical approach; however, there are still challenges in this practice. Kameda et al. informed that STAS might be detected in frozen portions with an accuracy of 80%, a specificity of 92.4%, and a sensitivity of 71%.^27^ However, those findings need to be verified.

In this study, there are some limitations. Firstly, this is a single‐center, small sample size (*n* = 130) retrospective study. Secondly, operations in the process of surgery and pathological section may affect STAS incidence. Further investigation is required since there is still disagreement about whether STAS is an in vivo impact in any instance or possibly an artifact.

In conclusion, more than half of the stage IB NSCLC tested positive for STAS. The STAS is an aggressive pathological feature. It has the potential to significantly reduce RFS and OS, while also functioning as an independent predictor of OS and RFS. STAS was significantly connected to poor differentiation and vascular invasion, and worse differentiation was STAS‐independent predictor. However, our results need to be further validated by a large‐sample, multicenter prospective study. Moreover, based on these findings, STAS should be included in the pathological report as it may assist clinicians in making better therapeutic strategy to improve prognosis. In addition, additional work is also needed to improve the effectiveness of STAS detection.

## AUTHOR CONTRIBUTIONS

**Zixuan Chen:** Data curation (equal); formal analysis (equal); investigation (equal); validation (equal); writing – original draft (equal); writing – review and editing (equal). **Xianqiao Wu:** Data curation (equal); formal analysis (equal); investigation (equal); validation (equal); writing – original draft (equal); writing – review and editing (equal). **Tianzheng Fang:** Data curation (equal); formal analysis (equal); investigation (equal); validation (equal); writing – original draft (equal); writing – review and editing (equal). **Zhen Ge:** Investigation (equal); methodology (equal); validation (equal); writing – review and editing (equal). **Jiayuan Liu:** Investigation (equal); methodology (equal); validation (equal); writing – review and editing (equal). **Qinglong Wu:** Investigation (equal); software (equal); validation (equal); visualization (equal); writing – review and editing (equal). **Lin Zhou:** Investigation (equal); software (equal); validation (equal); visualization (equal); writing – review and editing (equal). **jianfei Shen:** Conceptualization (equal); project administration (equal); resources (equal); supervision (equal); validation (equal); writing – review and editing (equal). **Chengwei Zhou:** Conceptualization (equal); project administration (equal); resources (equal); supervision (equal); validation (equal); writing – review and editing (equal).

## CONFLICT OF INTEREST STATEMENT

There are no declared conflicts of interest.

## ETHICS STATEMENT

This investigation has been authorized by the medical ethics committees of The First Affiliated Hospital of Ningbo University, and Taizhou Hospital of Zhejiang Province.

Project batch number is KS20232003. Project acceptance number is 2023KS0009.

## Data Availability

The data that support the findings of this study are not openly available due to sensitivity and privacy of human data but are available from the corresponding author on reasonable request.
